# Resistance to Dihydroartemisinin

**DOI:** 10.3201/eid1305.061442

**Published:** 2007-05

**Authors:** Eric Legrand, Beatrice Volney, Jean-Baptiste Meynard, Philippe Esterre, Odile Mercereau-Puijalon

**Affiliations:** * Institut Pasteur de la Guyane, Cayenne, French Guiana; † Institut Pasteur, Paris, France

**Keywords:** Plasmodium falciparum, artemether, resistance, Pfatpase6, French Guiana, letter

## To the Editor

The title of the letter by Cojean et al. (*1*) is misleading. The data presented essentially point to an absence of in vitro resistance to dihydroartemisinin (dhART) in the panel of African isolates studied, with 1 of 397 isolates having an elevated 50% inhibitory concentration (IC_50_) for dhART. The S769N *PfATPase6* mutation associated with in vitro resistance to artemether (*2*) was observed in 1 isolate. This mutant isolate had a low IC_50_ for dhART, but its IC_50_ for artemether has not been tested. Since the relationship between in vitro susceptibility to artemether and dhART is still uncertain (*3*), these data do not disprove the association of a *PfATPase6* S769N polymorphism with elevated IC_50_ for artemether that was observed in isolates from French Guiana (*2*).

Worth noting is that the association of the S769N *PfATPase6* polymorphism with elevated IC_50_ for artemether was confirmed in an isolate collected in French Guiana in 2005; that isolate had an IC_50_ for artemether of 127 nmol/L. Molecular typing identified 2 clonal types, 1 with a wild-type *PfATPase6* allele and 1 with a S769N single mutant. After 3 weeks of in vitro cultivation without drug, the mutant allele was no longer detected and the IC_50_ for artemether was 8.2 nmol/L. This finding suggests poor fitness of the mutant allele under standard culture conditions.

The observation of an additional case of in vitro resistance to artemether in French Guiana 3 years after the first cases is of concern. Reinforcement of surveillance is needed as is clarification of the relationship of in vitro susceptibility to artemether and artesunate, the derivatives currently included in artemisinin-based combination therapies (ACTs). Surveillance and clarification would be particularly timely since emerging clinical or parasitologic failures to some ACTs have been reported (*4*,*5*).
