# Supplementary material for: Resistance to Dihydroartemisinin
Source: Emerg Infect Dis. 2007 May;13(5):808–9. doi: 10.3201/eid1305.061442 (PMC2738458; doi:10.3201/eid1305.061442)
Supplement: Related texts

# Reply: In Response

**Authors:** Sandrine Cojean, Véronique Hubert, Jacques Le Bras, Rémy Durand

**Affiliations:** * Hôpital Bichat Claude Bernard, Paris, France; † Université Paris 5; Paris, France; ‡ Hôpital Avicenne, Bobigny, France; § Université Paris 13, Bobigny, France

The original title of our article was “Lack of *Plasmodium falciparum* in Vitro and Genomic Resistance to Dihydroartemisinin in Travelers Returning to France from Africa.” EID’s shortening of the title (*1*) led to the perception that the letter title was misleading, but it was not on purpose. We have recently tested the 50% inhibitory concentration for artemether of the S769N *PfATPase6* isolate that we had kept in liquid nitrogen, and it showed susceptibility.

We underline that the previously reported clinical or parasitologic failures to some artemisinin-based combination therapies (*2**,**3*) were not synonymous with the emergence of resistance to artemisinin compounds. In the study by Grandesso et al., a combination of artesunate plus amodiaquine was given to children <5 years of age who lived in an area in which amodiaquine alone was ineffective to adequately treat uncomplicated falciparum malaria in 1 of 3 cases at day 28 (*2*). Such a combination (artesunate plus amodiaquine) was nearly equivalent in 1 of 3 cases to a 3-day artesunate monotherapy, which may fail to completely cure children because of the short half-life of artesunate. In the study by Bukirwa et al., no recrudescence occurred in patients treated with artesunate plus amodiaquine and only 2 of 199 patients treated with artemether plus lumefantrine experienced recrudescence at day 28 (*3*). As Birkiwa et al. themselves acknowledged, artemether plus lumefantrine was not administered with food, and it is known that lumefantrine is absorbed better when it is taken with a small amount of fat. Thus, the clinical failures observed did not necessarily reflect *P. falciparum* resistance to artemisinin compounds.

## References

[1] Cojean S, Hubert V, Le Bras J, Durand R. Resistance to dihydroartemisinin. Emerg Infect Dis. 2006;12:1798–9.1728364510.3201/eid1211.060903PMC3372362

[2] Grandesso F, Hagerman A, Kamara S, Lam E, Checchi F, Balkan S, Low efficacy of the combination artesunate plus amodiaquine for uncomplicated falciparum malaria among children under 5 in Kailahun, Sierra Leone. Trop Med Int Health. 2006;11:1017–21. 10.1111/j.1365-3156.2006.01655.x16827702

[3] Bukirwa H, Yeka A, Kamya MR, Talisuna A, Banek K, Bakyaita N, Artemisinin combination therapies for treatment of uncomplicated malaria in Uganda. PLoS Clin Trials. 2006;1:e7. 10.1371/journal.pctr.001000716871329PMC1488893
